# Supplementary material for: Effects of standard and low doses of estradiol on markers of endometrial receptivity in frozen-thawed embryo transfer cycles: Double-blind, randomized clinical trial
Source: PLoS One. 2025 Jul 28;20(7):e0328871. doi: 10.1371/journal.pone.0328871 (PMC12303338; doi:10.1371/journal.pone.0328871)
Supplement: S1 — (DOCX) [file pone.0328871.s001.docx]

# **Article Title: Effects of Standard and Low Doses of Estradiol on Markers of Endometrial Receptivity in Frozen-thawed Embryo Transfer Cycles: Double-blind, Randomized Clinical Trial**

**Study Protocol**

**Study Design**
This is a double-blinded, randomized clinical trial.

**Study Population**
Fifty infertility patients were scheduled for frozen-thawed embryo transfer in the in vitro fertilization (IVF) program at the Infertility Clinic, Faculty of Medicine, Siriraj Hospital, Bangkok, Thailand.

**Inclusion Criteria**

1. Patients scheduled for frozen-thawed embryo transfer in the IVF program
2. Women aged between 18 and 45 years
3. Normal follicle-stimulating hormone (FSH), luteinizing hormone (LH), and estradiol levels during the early follicular phase
4. Regular menstrual cycles for at least three months prior to enrollment
5. Provided written informed consent after comprehensive counseling

**Exclusion Criteria**

1. Contraindications to estrogen use (e.g., endometrial cancer, unexplained bleeding, thromboembolism, or liver disease)
2. Uterine cavity abnormalities (e.g., synechiae or polyps)
3. Use of estrogen within the three months prior to enrollment

**Study Procedure (Figure 1)**

1. **Baseline Data Collection**:
   After providing informed consent, demographic data were collected, including age, body mass index, history of live birth and miscarriage, curettage history, and infertility duration.
2. **Randomization**:
   Patients were randomly assigned to either the low-dose or standard-dose estradiol hemihydrate (Estrofem) groups using computer-generated randomization, with 25 patients per group. Allocation concealment was achieved using sealed envelopes, ensuring both the patients and investigators were blinded to treatment assignments.
3. **Treatment Protocol**:
   - **Low-dose group**: Patients took 4 mg/day of Estrofem, consisting of two 1-mg capsules, one taken in the morning and one in the evening.
   - **Standard-dose group**: Patients took 6 mg/day of Estrofem, consisting of one 1-mg capsule and one 2-mg capsule in both the morning and evening. Both capsule types looked identical.
   - Treatment commenced on day 2 of the menstrual cycle and continued for 10 days.

**First Follow-Up (Day 12 of the Menstrual Cycle)**

1. Endometrial thickness was assessed via transvaginal ultrasonography (Xario 100; Toshiba Medical Systems, Japan) by a single experienced gynecologist who was blinded to the treatment allocation.
2. Blood samples were collected to measure estradiol and progesterone levels.
3. Medication compliance was verified by counting the returned capsules in each patient's medication pack.
4. Participants started taking micronized progesterone (Utrogestan), 600 mg per day, divided into three doses, for seven consecutive days.

**Second Follow-Up (Day 19 of the Menstrual Cycle)**

1. Blood samples were collected again to measure estradiol and progesterone levels.
2. An endometrial biopsy was performed using the Wallach Endocell endometrial sampling device (CooperSurgical, USA).
3. The collected endometrial tissue was immediately placed in a tissue cassette, fixed in 10% neutral-buffered formaldehyde overnight for paraffin embedding, and prepared for subsequent immunohistochemical analysis.

**Immunohistochemical Staining Protocol**

1. After the endometrial biopsy, specimens were immediately fixed in 10% neutral-buffered formaldehyde and embedded in paraffin wax.
2. The paraffin-embedded tissues were sectioned into 3-µm slices and mounted onto SuperFrosted Plus adhesion slides (Thermo Scientific, UK).
3. The sections were incubated overnight at 60 °C.
4. Immunostaining was performed using the Ventana BenchMark Ultra Staining System (N750-BMKU-FS 05342716001; Roche) following an optimized protocol to detect the expression of the regulatory proteins HOXA-10, HOXA-11, and integrin αvβ3.
5. For HOXA-10 detection, antigen retrieval was conducted at 95 °C for 64 minutes in OptiView CC1 (pH 6.0), followed by incubation with rabbit polyclonal anti-human HOXA-10 primary antibody (GTX37412, GeneTex) at a 1:50 dilution at 37 °C.
6. HOXA-11 and integrin αvβ3 were detected with a milder protocol: antigen retrieval was performed using Mild Ultra CC1 (pH 6.0) for 52 minutes, followed by incubation with rabbit polyclonal anti-human HOXA-11 (GTX48983, GeneTex, 1:200 dilution) and mouse monoclonal anti-human integrin αvβ3 (MAB1976, Merck, 1:1500 dilution) at 37 °C.
7. Detection was carried out using the Ultraview Universal DAB Detection Kit.
8. The sections were counterstained with hematoxylin, mounted using Permount solution, and secured with coverslips.
9. The staining process and analysis for HOXA-10, HOXA-11, and integrin αvβ3 expression were standardized before assessment.
10. Control slides containing normal secretory-phase endometrial epithelium were used as positive controls, while slides processed without primary antibodies served as negative controls.

**Immunohistochemical Staining Analysis**

1. The intensity of immunohistochemical staining for HOXA-10, HOXA-11, and integrin αvβ3 was quantified using intensity scores in ImageJ 1.46r/Java 1.6.0_20 (64-bit, USA).
2. Staining analysis focused on the glandular epithelium of each section and was independently performed by two observers.
3. Final intensity scores for each protein were obtained by averaging the scores assigned to each section.

**
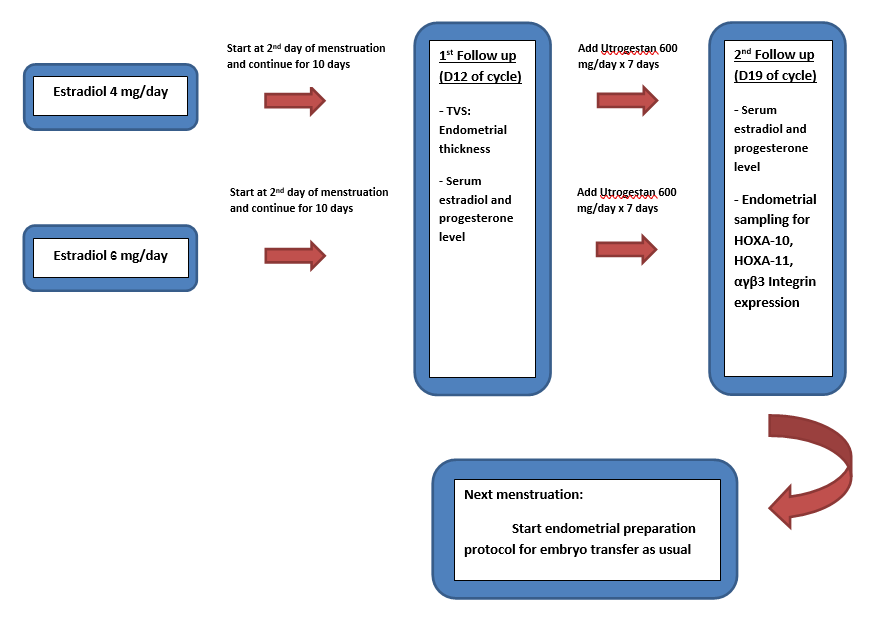
**

**Figure 1.** Diagram for Research Protocol
