# Supplementary material for: Effects of standard and low doses of estradiol on markers of endometrial receptivity in frozen-thawed embryo transfer cycles: Double-blind, randomized clinical trial
Source: PLoS One. 2025 Jul 28;20(7):e0328871. doi: 10.1371/journal.pone.0328871 (PMC12303338; doi:10.1371/journal.pone.0328871)
Supplement: Attachment — Submitted filename: Response_to_Reviewer_Comments_auresp_4.docx [file pone.0328871.s006.docx]

**Response to Reviewer Comments (30062025)**

**Reviewer #1:** It is now clear to readers that HOXA-10 is the sole gene selected as the primary outcome. However, the justification provided for its use in the sample size calculation—namely, that "this gene demonstrated the largest effect size and required the greatest number of participants" (lines 204–205)—is incorrect. In fact, detecting a larger effect size typically requires fewer, not more, participants. This rationale should be corrected before the manuscript can be accepted.

**Answer:** Thank you for your comment. Your comment is very useful and I totally agree with you. I have corrected as your comment.

The sample size calculation was based on the previous study (17). The sample size was calculated based on HOXA-10. HOXA-10 functions as an upstream master regulator, controlling the expression of other key genes involved in uterine development and endometrial receptivity (17).

**(mentioned in line 203-205).**
