# Supplementary material for: Effects of standard and low doses of estradiol on markers of endometrial receptivity in frozen-thawed embryo transfer cycles: Double-blind, randomized clinical trial
Source: PLoS One. 2025 Jul 28;20(7):e0328871. doi: 10.1371/journal.pone.0328871 (PMC12303338; doi:10.1371/journal.pone.0328871)
Supplement: Attachment — Submitted filename: Response to Reviewer Comments PLOS ONE 08042025.docx [file pone.0328871.s004.docx]

**Response to Reviewer Comments**

**Reviewer #1:**

**1. Authors have added in sample size justification (lines 202-205). However, key estimates used in such sample size calculation such as effect size and corresponding statistical test are missing. In addition, Lines 109-110 said that the primary outcome is the expression levels of three genes. So multiple testing adjustment should be considered in the sample size calculation.**

**Answer:** Thank you for your suggestion. In the study, the appropriate statistical test is the Independent samples t-test. The effect size is measured using Cohen’s d: (Mentioned in line 205-206)

Moreover, multiple testing adjustment was actually considered in the sample size calculation and sample size for HOXA-10 was the greatest number. So, authors used this sample size as a reference number for sample size calculation.

**2. There is still no report about study compliance. Lines 145-146 described how study compliance was recorded. Were all 48 patients in the final analysis compliant to study protocol?**
**Answer:** Thank you for your comment. Indeed, all 48 patients in the final analysis were compliant to study protocol. But in the low-dose group, one patient’s data were excluded due to a diagnosis of endometrioid adenocarcinoma of the uterus identified in the final pathological report. In the standard-dose group, data from one patient were discarded due to incorrect drug consumption. Therefore, the analyses were performed on a per-protocol basis (24 patients in each group) (Mentioned in line 209-213)

**3. All p-values in Table 1 have been removed but Line 214 still added a p-value for comparing age between two dose groups.**

**Answer:** Thank you for your comment. P-value were deleted in line 216-217.

**Reviewer #2: (No Response)**

**Reviewer #3: I apologize for lack of specificity in my comment to be address. My concern is that Hoxa10 and Hoxa11 are observed in the cytoplasm, which is an unusual location for a transcription factor that may have a functional role in the endometrium. The references you site for expression of these genes in the endometrium (17, 18, and 26) report both cytoplasmic and nuclear localization. Can you could comment on the functional significance for Hoxa10 and Hoxa11 being localized in the cytoplasm?**

**Answer:** Thank you for your comment. I agree that Hoxa10 and Hoxa11 are transcription factors mainly located in the nucleus. However, the result in the study showed the expression of Hoxa10 demonstrated mainly in the cytoplasm of the glandular epithelial area, the Hoxa10 protein is localized in the nucleus of stroma cells. These findings are similar to the study from Yang 2017 (17). The function of these transcription factors in the cytoplasm, however, remained unclear but might be related to the post-transcription process, such as the regulation of protein synthesis. We added in the discussion in line 286-292.
