# Supplementary material for: Effects of standard and low doses of estradiol on markers of endometrial receptivity in frozen-thawed embryo transfer cycles: Double-blind, randomized clinical trial
Source: PLoS One. 2025 Jul 28;20(7):e0328871. doi: 10.1371/journal.pone.0328871 (PMC12303338; doi:10.1371/journal.pone.0328871)
Supplement: Attachment — Submitted filename: Response to Reviewer Comments.docx [file pone.0328871.s005.docx]

**Response to Reviewer Comments (13062025)**

**Reviewer #1: It appears that the authors are still unclear about the application of multiple testing adjustment. The current version of the manuscript states that a type I error rate of 0.05 was used for the sample size calculation, which does not indicate that any correction for multiple comparisons was applied. Additionally, the authors mention that the sample size was based on HOXA-10, the gene requiring the largest sample, suggesting that different effect sizes were assumed for the three genes. These assumptions, along with the rationale for using different effect sizes across genes, should be explicitly described and justified in the main text.**

**Answer:** Thank you for your comment. I agree with you.

The current version of the manuscript states that a type I error rate of 0.05 was used for the sample size calculation. This is because we defined one primary outcome (HOXA-10) and treat the others as secondary for calculation. So, we used alpha = 0.05 for that one outcome in sample size calculation. We’ve already revised the primary and secondary outcomes as in **line 54-56 & 109-113.**

Additionally, the sample size was calculated based on HOXA-10, as this gene demonstrated the largest effect size and required the greatest number of participants. HOXA-10 functions as an upstream master regulator, controlling the expression of other key genes involved in uterine development and endometrial receptivity **(mentioned in line 203-206).**
